# Seasonal characteristics of storms over the Indian subcontinent

**DOI:** 10.1038/s41598-021-82237-w

**Published:** 2021-02-08

**Authors:** Kapil Dev Sindhu, G. S. Bhat

**Affiliations:** Centre for Atmospheric and Oceanic Sciences, Indian Institute of Science, Bangalore, 560012 India

**Keywords:** Climate sciences, Atmospheric science

## Abstract

Storms are convective cells responsible for the major fraction of convective precipitation. Here, the pre-monsoon and monsoon season characteristics of storms are reported at Lucknow, Patna, Bhopal, and Nagpur in India using equivalent radar reflectivity factor ($\hbox {Z}_e$) given at these radar locations. It is observed that the lifetime, speed of propagation, area, volume, echo top height and thickness lie in ranges 0.3–3 h, 5–60 km $\hbox {h}^{-1}$, 4–184 $\hbox {km}^2$, 8–1600 $\hbox {km}^3$, 2–14 km, and 0.5–16 km respectively. For both seasons, the relationships between radar estimated rain volume (RERV; range $10^4$–$10^7$ $\hbox {m}^3$) and area-time integral (ATI; range 1–100 $\hbox {km}^2$ h) are established which are considered as a representative of total precipitation resulted from an individual storm during its life cycle. The results from statistical analysis of RERV-ATI pairs suggest that storms at Lucknow have similar seasonal characteristics at 87% confidence interval while other locations exhibit dissimilarities. In addition, the vertical profiles of radar reflectivity (VPRRs) of storms are constructed at their life phases, namely cumulus, mature and dissipation. It is concluded that the vertical $\hbox {Z}_e$ gradient in mixed-phase region (5–8 km) is lower (2–2.9 dBZ $\hbox {km}^{-1}$) at cumulus and dissipation phases than at mature phase (3.6–4.4 dBZ $\hbox {km}^{-1}$) in monsoon season. For pre-monsoon season, this gradient lies between 3.3–5.2 dBZ $\hbox {km}^{-1}$ at mature phase. Our results are of great importance for advancing knowledge about storm-scale, which has implications in short-range weather forecasting as well as developing new convective parametrization schemes.

## Introduction

**Figure 1:**
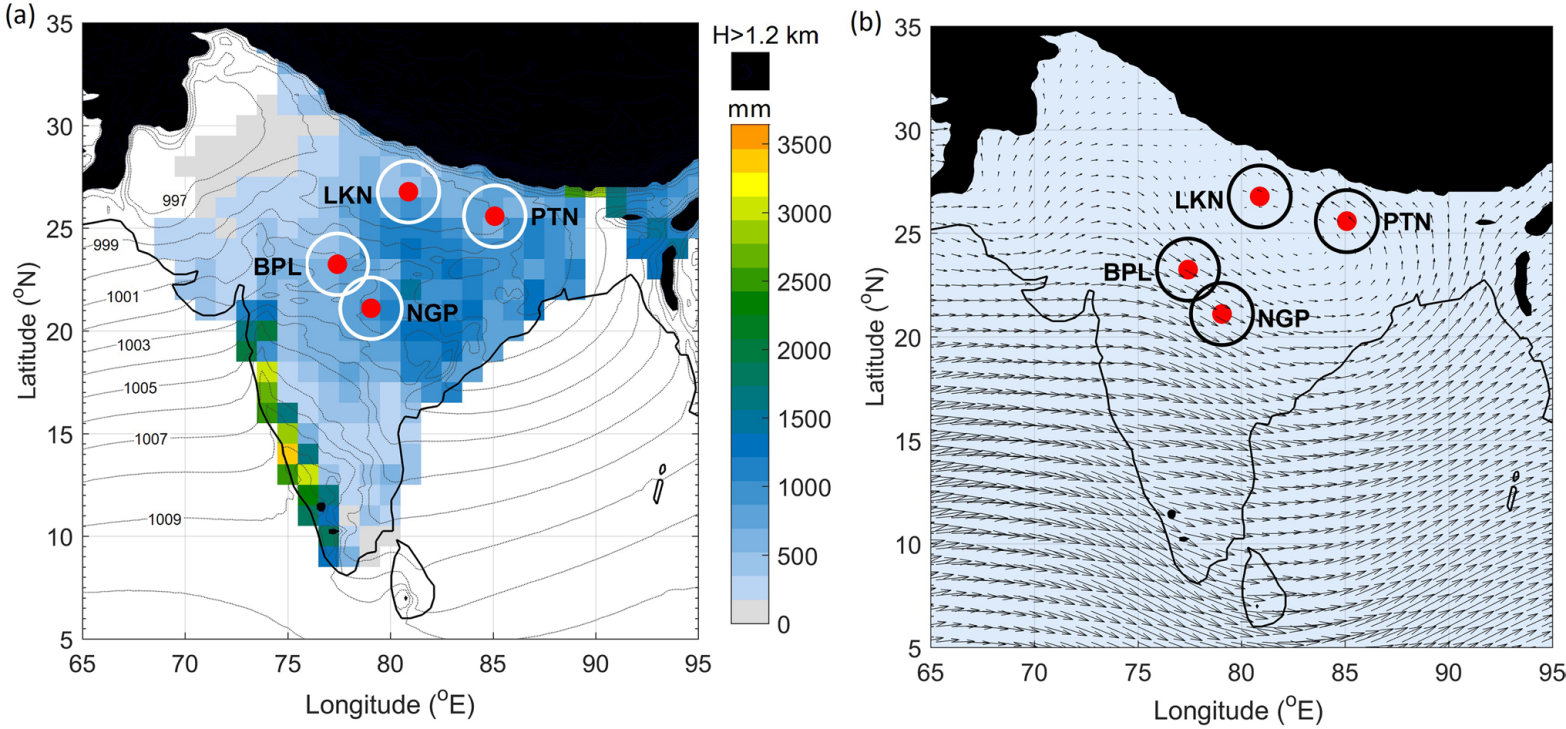
Geographical locations of S-band Doppler weather radars included in the study: (1) Lucknow (LKN; 26.77$^{\circ }$ N, 80.88$^{\circ }$ E, 143 m), (2) Patna (PTN; 25.58$^{\circ }$ N, 85.08$^{\circ }$ E, 67 m), (3) Bhopal (BPL: 23.24$^{\circ }$ N, 77.42$^{\circ }$ E, 570 m), (4) Nagpur (NGP; 21.09$^{\circ }$ N, 79.06$^{\circ }$ E, 335 m). (**a**) June to September average rainfall (mm) based on 1$^{\circ }$ $\times$ 1$^{\circ }$ gridded rainfall dataset of IMD for the period 1951–2003 is overlaid. The curves onto rainfall patterns are the mean sea level (in hPa) pressure from the ERA5 dataset for July during the years 1979–2018. Grids with surface elevation $>1200$ m are masked as shown in the black patch. The outer circle around each DWR location shows a 150 km radar range. (**b**) Average wind pattern at 850 hPa for the June–September months of 2016.

Embedded within a mesoscale convective system (MCS)^1^, there are a few to several intense convective cells, henceforth called storms^2^. These storms lie in scale C (1–10$^2$ $\hbox {km}^2$) and D (10$^2$ and 10$^3$ $\hbox {km}^2$) categories of atmospheric weather systems^3^. Storms consist of a group of closely spaced cumulonimbus (Cb) clouds and contribute more than 90% of the total convective precipitation associated with an MCS^2^. Cb clouds are responsible for the vertical transport of momentum and energy from the boundary layer to the upper troposphere^4,5^. A convective cloud may be thought of as a diabatic plume^6^, and the horizontal size of the plume matters for lateral entrainment^7,8^. It is rare to find a single Cb cloud in the tropical atmosphere. Nature seems to favour several of them growing together in the form of a storm, making the lateral size of a storm relevant to the lateral entrainment. Storm size/area also becomes relevant in remote sensing of precipitation, particularly in deciding the size of the horizontal footprint of the payload since more than 90% of convective precipitation results from storms. Therefore, for both scientific and practical purposes, knowing storm characteristics, including storm size, is important. A three-dimensional field of equivalent radar reflectivity factor ($\hbox {Z}_{ e}$^9^) is used to detect storms^10,11^. A storm is defined as a group of connected cloudy pixels having their $\hbox {Z}_{ e}$ and total volume above some specified thresholds^11^. Hereafter, a pixel having its $\hbox {Z}_{ e}$ above the noise threshold of the radar is called as a cloudy pixel. $\hbox {Z}_{ e}$ thresholds between 25 and 45 dBZ and volume thresholds between 10 and 50 $\hbox {km}^3$ have been adopted in a majority of previous studies^11–16^. Derived storm characteristics depend on the thresholds used. Therefore, we associate the $\hbox {Z}_{ e}$ threshold with a storm while describing its properties hereafter. For example, a 30-dBZ storm is defined using the $\hbox {Z}_{ e}$ threshold of 30-dBZ. Several studies have reported characteristics (e.g., area, height, propagation speed, etc.) of storms^12,14,16,17^. Typically, storms have lifetimes of an hour or less (only a few exceed 3 h^18,19^). Over the Sydney region, Potts et al.^12^ found that 30-dBZ storm’s maximum echo-top height lies between 2 and 16 km. During the Australian monsoon season, 35-dBZ storms over the Darwin area have lifetimes of less than 1 hour and their heights lie between 3 and 18 km with a mean height of 10 km^13^. Goudenhoofdt and Delobbe^14^ studied the 40-dBZ storms (with a volume threshold of 10 $\hbox {km}^3$) over Belgium using C-band radar data. The area of these storms varied between 2 and 100 $\hbox {km}^2$ and they had a median propagation speed of 30 km $\hbox {h}^{-1}$. Novo et al.^17^ reported that the maximum echo-top heights of 25-dBZ storms over Cuba lie between 2 and 18 km. Shah et al.^16^ estimated that the areas of 28-dBZ storms over Italy are less than 200 $\hbox {km}^2$. Sindhu and Bhat^2^ studied 30-dBZ storm properties embedded within monsoonal cloud system (CS, defined as a set of connected cloudy pixels having an area of at least 1600 $\hbox {km}^2$ and containing at least one pixel with $\hbox {Z}_{ e}$ $>30$ dBZ) at 4 locations in India (Kolkata, Hyderabad, Nagpur, and Patiala). According to this study, average echo-top heights of 30-dBZ storms are found between 6 and 10 km while average areas of storms are observed between 25 and 175 $\hbox {km}^2$^2^. Sindhu and Bhat^19^ quantified the properties of 30-dBZ and 40-dBZ storms over New Delhi, a northwest region of the Indian subcontinent. The average propagation speed of these storms varied in range 5–65 km $\hbox {h}^{-1}$. The maximum altitude and area of 30-dBZ storms exceeded 17 km and 300 $\hbox {km}^2$ respectively. Storm studies carried out so far cover a limited number of areas and weather conditions. For example, storm properties during the pre-monsoon season are missing over the Indian region. As stated in the opening paragraph earlier, there is a need to understand storm characteristics in more detail and covering different climatic conditions. Atmospheric conditions (e.g., temperature, humidity, and wind fields) are distinctly different between pre-monsoon and monsoon seasons^20^. An intercomparison of characteristics of storms during these two periods may bring out the influence of environmental factors on storm characteristics. Under the ‘Interaction of Convective Organization and Monsoon Precipitation, Atmosphere, Surface and Sea (INCOMPASS)’ field campaign (an Indo-UK joint project on south Asian monsoon^21^), the India Meteorological Department (IMD) made available its radar data for May to September months of the year 2016 for research purpose. The present study is based on IMD radar data at four locations (Fig. 1) and with the following objectives.

1. Study of 40-dBZ storms’ life characteristics,
2. Differences in storms’ characteristics between monsoon and pre-monsoon seasons.

## Data and methods

### Data

The primary data used in this work is equivalent radar reflectivity factor ($\hbox {Z}_{ e}$). It is proportional to the 6th power of hydrometeor size, a proxy for hydrometeor concentration and rain rate (R) in a precipitating cloud. Both $\hbox {Z}_e$ and *R* are related as *Z*=148*R*$^{1.55}$ for convective echoes^22^. The $\hbox {Z}_{ e}$ data collected with S-band Doppler weather radars (DWRs) of IMD at Lucknow (LKN), Patna (PTN), Bhopal (BPL), and Nagpur (NGP) located over the Indian subcontinent are analysed in the present study (Fig. 1). The geo-locations and technical specifications of these DWRs are given in Table 1. The mean sea level pressure shown in Fig. 1 is that of the July month based on the fifth generation of ECMWF atmospheric reanalysis dataset (ERA5, Hersbach et al.^23^) for the period 1979–2018. The mean of rainfall during the monsoon season for the period 1951–2003 over India is IMD’s rain gauge based gridded rainfall dataset. Nagpur and Bhopal are situated within the main monsoon zone^24^ while areas under Lucknow and Patna radars’ coverage lie between low-land regions (0–300 m) and foothills (300–3000 m) of the Himalayas and juxtaposed to the monsoon zone (Fig. 1a). The mean wind pattern at 850 hPa pressure level suggests that Lucknow and Patna encounter southeasterly winds while Bhopal and Nagpur encounter northwesterly winds during monsoon season (Fig. 1b). Lucknow and Patna areas are among regions of the globe that experience wide intense convective echoes (area of 40 dBZ echo $>1000$ $\hbox {km}^2$), and large stratiform areas ($>50,000$ $\hbox {km}^2$)^25^. CSs at Nagpur are dominated by stratiform precipitation^2^. Bhopal and Nagpur are in the paths of monsoon depressions and low-pressure systems^26^. IMD’s radar data follow the calibration procedure given by IMD^27^. The data is passed through additional quality checks for noise removal^2^. Radar data within a 150 km radar range is included in the study to avoid super refraction and beam widening effects^28,29^. $\hbox {Z}_{ e}$ data below an altitude of 1 km are likely to be contaminated due to multiple reflections from the earth’s surface^30^, and are excluded. Raw $\hbox {Z}_{ e}$ data are re-gridded to Cartesian-coordinate grids with a pixel size of 2 km $\times$ 2 km in the horizontal and 0.5 km in the vertical using the Radx2Grid subroutine of Radx-algorithms developed in the Research Applications Laboratory, National Centre for Atmospheric Research, USA^31^. The altitude in the Cartesian-gridded data is in meters above ground level (AGL). The typical S-band radar’s accuracy is $\sim$ 5 dBZ at 150 km range, and to avoid noisy data affecting the results, $\hbox {Z}_{ e}$ values $<10$ dBZ have been discarded. The sounding data is obtained from the IMD upper air observational network to assess the environmental conditions in pre-monsoon and monsoon seasons.

**Table 1:** Geographical details and technical specifications of S-band DWRs located over the Indian subcontinent. The abbreviations Lat, Lon, and Alt refer to latitude, longitude, and altitude respectively. Here, $\hbox {N}_{mo}$ and $\hbox {N}_{pm}$ indicate the number of storms detected in monsoon and pre-monsoon seasons respectively.

Specifications	Lucknow	Patna	Bhopal	Nagpur
Lat, Lon, Alt	26.77$^{\circ }$ N, 80.88$^{\circ }$ E,143 m	25.58$^{\circ }$ N, 85.08$^{\circ }$ E, 67 m	23.24$^{\circ }$ N, 77.42$^{\circ }$ E, 570 m	21.09$^{\circ }$ N, 79.06$^{\circ }$ E, 335 m
Wavelength	10.43 cm			
Scan elevations	0.22$^{\circ }$/0.54$^{\circ }$, 0.99$^{\circ }$, 1.99$^{\circ }$, 2.9$^{\circ }$, 4.5$^{\circ }$, 5.9$^{\circ }$, 8.87$^{\circ }$/9.01$^{\circ }$, 12$^{\circ }$, 16$^{\circ }$, 21$^{\circ }$			
Azimuths	0$^{\circ }$–359$^{\circ }$			
Gate size	500 m	250 m	500 m	500 m
Beamwidth	1$^{\circ }$			
Total radar volumes	5793	14346	11923	7910
$\hbox {N}_{mo}$ ($\hbox {N}_{pm}$)	1555 (17)	1592 (717)	4477 (836)	303 (318)

### Storm identification

**Figure 2:**
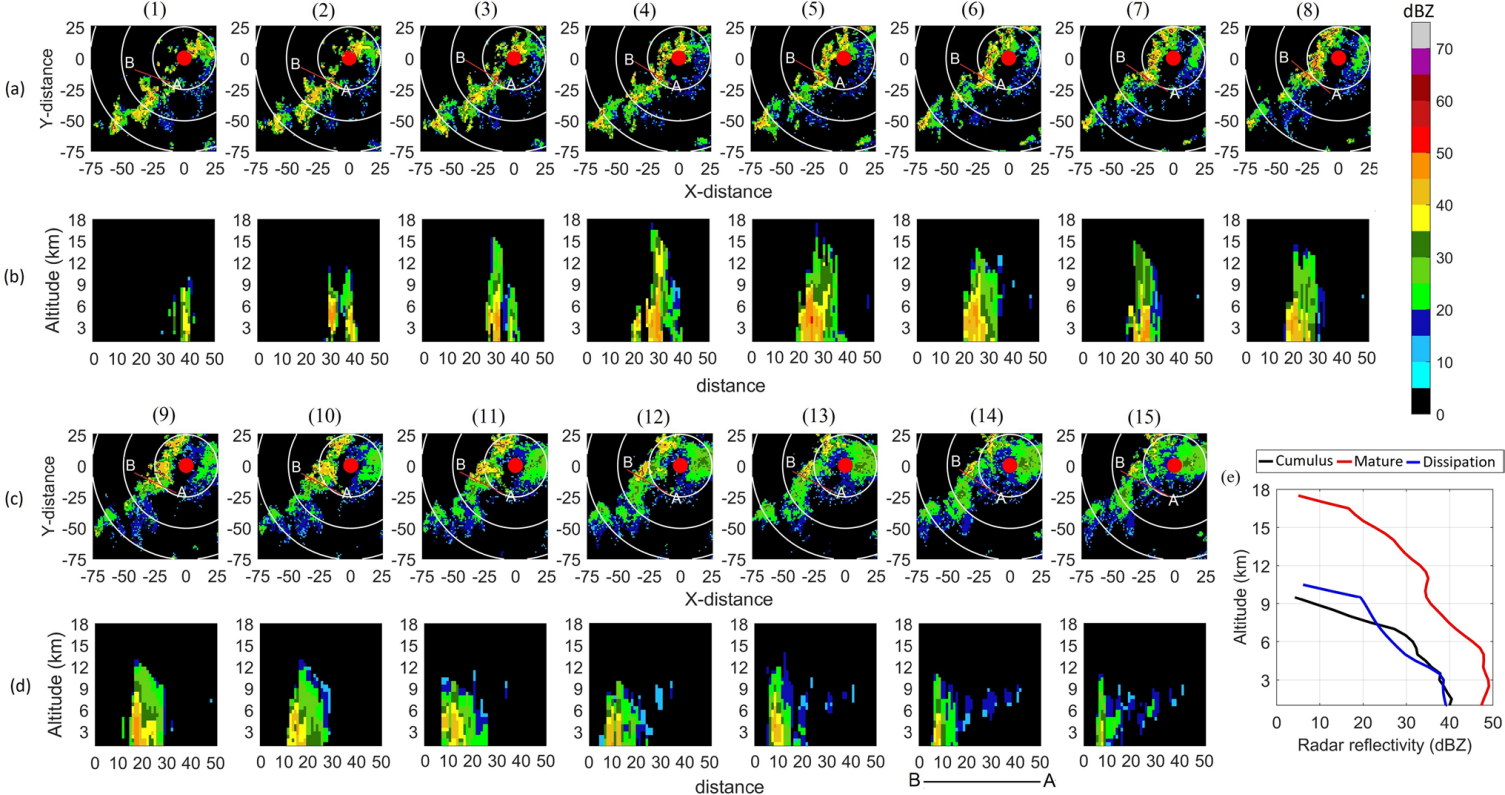
Temporal evolution of a storm observed on 17 September 2016 over the Bhopal region before the cumulus phase (1510 IST, top left panel) till the time instant after the dissipation (1732 IST, bottom most right) phase. The panels in rows (**a**) and (**c**) are the composite reflectivity patterns, and rows (**b**) and (**d**) are the vertical sections taken across the storm along line BA. Successive panels from left to right are 10 min apart. Panel (**e**) shows the VPRRs at cumulus, mature, and dissipation phases. The color bar is $\hbox {Z}_{ e}$ in the dBZ unit.

The evolution of convection captured by the radar at Bhopal on 17 September 2016 is shown in Fig. 2. This was an afternoon system. Successive frames are 10 minutes apart, starting at 1510 IST. To gain insight into the development of precipitation, we show vertical sections taken along line B–A whose position is changed with time. X-distance shown in panels (b) are in grid units (gu, and 1 gu = 2 km) measured from B to A. In this work, the intensity of a convective cell is based on the maximum $\hbox {Z}_{ e}$ present. If maximum $\hbox {Z}_{ e}$ exceeds 40 dBZ, then it is termed intense while less intense cell means peak reflectivity is below 40 dBZ. Similarly, we call a storm an intense storm if defined using a threshold of 40 dBZ or higher while those based on 35 dBZ and lower thresholds are less intense storms. At the first instant (Frame 1), a precipitating Cb cloud with its vertical extent penetrating 10 km height is observed at 40 gu. The maximum $\hbox {Z}_{ e}$ is less than 40 dBZ and occurs between 3 and 6 km levels. There are also signs of another convective cell between 30 and 40 gu. 10 minutes later (Frame 2), cell at 40 gu has grown in the vertical (crossing 12 km level) and also intensified with $\hbox {Z}_{ e}$ exceeding 40 dBZ in the lower and mid-troposphere. This cell is tilted and leans towards B in the upper troposphere. A cell around 30 gu which is faintly visible in Frame 1 has developed in the vertical and its maximum $\hbox {Z}_{ e}$ exceeds 40 dBZ and lies between 4 and 6 km. In Frame 3, the cell at 40 gu is weakening while that at 30 gu has intensified further, its vertical extent has crossed 15 km level and the region with $\hbox {Z}_{ e}>40$ dBZ has descended below 3 km. Cells at 30 and 40 gu have started merging in the mid-troposphere while maintaining distinct identities below 3 km. In Frame 4, the cell at 30 gu has grown to almost 18 km in the vertical, and the region with $\hbox {Z}_{ e}>40$ dBZ has expanded laterally. The Cells at 30 and 40 gu have merged giving a larger area under high precipitation at lower levels, and a new cell is developing near 20 gu. In Frame 5, cells at 20 and 30 gu have merged, and maximum $\hbox {Z}_{ e}$ exceeds 50 dBZ and occurs between 4 and 5 km levels. Frames 1–5 shows that most intense precipitation develops in the mid-tropospheric region and then descends to the lower levels. By the time of Frame 9, cell between 20 and 30 gu is dissipating while new ones are developing between 10 and 20 gu which merge in Frame 10. Signs of developing anvil region is observed going from Frame 11 to 15. Convective cells have dissipated by the time of Frame 15. It is clear from Fig. 2 that the measured spatial extent and life span of a storm depend on the thresholds specified while defining the storm. At lower values of the threshold (e.g., 25 or 30 dBZ), the merging of storms becomes more likely which increases its life span as well. On the other hand, storms defined with $\hbox {Z}_{ e}$ thresholds of 40 dBZ, will be smaller in spatial extent with a shorter life span. In the present study, we have selected a $\hbox {Z}_{ e}$ threshold of 40 dBZ and a volume threshold of 25 $\hbox {km}^3$. In the literature, a $\hbox {Z}_{ e}$ value of 40 dBZ is always associated with convective precipitation^22,32^. A 40-dBZ storm is intense and extreme rain events are invariably associated with cloud systems having such intense convective cells. One objective of the present work is to get insights into the nature of intense convective cells in the monsoonal cloud systems, hence the 40 dBZ threshold is chosen. Some characteristics of 30-dBZ storms have been reported in Sindhu and Bhat^2^. It may be noted that monsoonal precipitating clouds over the Indian sub-continent have properties between pure continental (which are intense) and pure maritime (which are less intense) systems^25^. Houze et al.^25^ used 40-dBZ $\hbox {Z}_{ e}$ reaching 10 km level (based on TRMM-PR data) to define the intense convective core. Although the definitions of intense core^25^ and storm (this work) are different, this work gives information on 40-dBZ convective cells and compliments their work. The development of convective cells observed in Fig. 2 follows the life cycle of thunderstorms described by Byers and Braham^33^, i.e., cumulus, mature, and dissipation phases. Since the S-band radar detects precipitation and not clouds, when a convective cell is first detected, the cloud is perhaps past its early cumulus phase. So, when we say the cumulus phase of a storm here, it means radar has detected a growing cell having at least 1 gu of 40 dBZ for the first time and that is taken as the cumulus phase (Fig. 2, Frame 2). When the vertical extent of a cell reaches the maximum, the cell is in its peak mature phase (Fig. 2, Frame 5). Decreased vertical extent and reduced peak $\hbox {Z}_{ e}$ are signs of the dissipation phase (Fig. 2, Frame 15). Storms are tracked using TITAN (Thunderstorm Identification, Tracking, Analysis, and Nowcasting), a set of subroutines developed at RAL, NCAR^11^. The storms are tracked only in the convective echoes identified by TITAN-algorithm. For this purpose, we used a fixed threshold of 40 dBZ. TITAN-algorithm uses an optimization technique to get the successor to the previously detected storm. The minimum overlap between two successive volume scans is set to two reflectivity pixels in the composite reflectivity pattern. The maximum allowable speed for the tracking storm is set to an arbitrary value of 100 km $\hbox {h}^{-1}$. If a time gap in between volume scans exceeds 20 minutes, then the previous storm track is terminated, and a new track is initiated. The full detail about storm-tracking procedures can be found at TITAN generated parameter file and its webpage https://ral.ucar.edu/projects/titan/home/storm_tracking.php. Properties of storms are extracted at each time instant during their lifetimes starting from the first detection till the last detection within the radar range of 150 km. The averaged properties of storms over their lifetimes (called ‘aggregate properties’) are also estimated.

### Area, echo-top height, thickness, radar estimated rain volume, and area-time integral

The storm area is defined as the area of the connected pixels contained in the 2-dimensional pattern of composite reflectivity (maximum $\hbox {Z}_{ e}$ of all altitude levels) of the storm. The echo-top height (ETH) of the storm is the maximum height of 40-dBZ echo detected in the storm. The storm thickness is calculated as the maximum echo-top height minus minimum echo-base height in the identified storm. The radar estimated rain volume (RERV; units: $\hbox {m}^3$) is defined as rain rate (R; units: mm h^−1^) integrated over the area (A; units: $\hbox {km}^2$) of connected $\hbox {Z}_{ e}$ pixels contained in a CAPPI (constant altitude plan position indicator, defined as horizontal cross section of radar volume scan data at constant altitude) at 1.5 km altitude of the storm during its lifetime (${ \tau }$; units: h)^34^. The expression for RERV is given by,

$$\begin{aligned} RERV=\int _{\tau }\int _{A} R dA dt \end{aligned}$$

Here, *R* is calculated at each time instant using the *Z*–*R* relationship (*Z*=148*R*$^{1.55}$) for convective echoes from Iguchi et al.^22^ and incorporated in the TITAN-algorithm, and *dt* is the time difference between two radar volume scans ($\sim$ 10 min) which is fixed in all calculations. The area-time integral (ATI) is a term that combines spatial extent and life span of a precipitation event^34^ and is defined by

$$\begin{aligned} ATI=\int _{i}A_{i} dt \end{aligned}$$

where *A*$_i$ is the *A* at *i*th time instant. The relationship between RERV and ATI is approximated by a power-law given by

$$\begin{aligned} RERV=K(ATI)^b \end{aligned}$$

where ${ K}$ and ${ b}$ are the coefficient and exponent respectively. The typical values of ${ K}$ are in the range of 10$^3$–10$^4$ and ${ b}$ is close to 1^35^.

### Vertical profile of radar reflectivity (VPRR)

Zipser and Lutz^36^ proposed the idea of ‘vertical profile of radar reflectivity (VPRR)’ that gives the most intense precipitating cloud possible in a given radar volume. We constructed VPRR taking a box of 10 km $\times$ 10 km in the horizontal (Zipser and Lutz 1994 used 8 km $\times$ 8 km) and considering all vertical levels having a valid $\hbox {Z}_{ e}$. The detailed procedure can be found in Zipser and Lutz^36^. While calculating average VPRR, any altitude level which has the sample size less than 10% of the maximum sample size among all altitude levels, is omitted.

### Sounding variables and environmental indices

The radiosonde measures several environmental variables at different atmospheric pressure levels. The present study includes the vertical profiles of the following sounding variables: environmental temperature ($\hbox {T}_e$), dewpoint temperature ($\hbox {T}_d$), and relative humidity (RH). The derived environmental indices include convective available potential energy (CAPE), convective inhibition energy (CINE), severe weather threat (SWEAT) index, and K-index.

CAPE is an integrated effect of the positive buoyancy of the rising undiluted parcel relative to its environment^37^. It is defined as follows:

$$\begin{aligned} CAPE=\int _{LFC}^{LNB}(T_{vp}-T_{ve}) R_{d}dln P \end{aligned}$$

where $\hbox {T}_{vp}$ and $\hbox {T}_{ve}$ are the virtual temperatures of the parcel and the environment, respectively. LFC is the level of free convection, and LNB is the level of neutral buoyancy, respectively.

CINE is represented by the negative area on the tephigram at the lowest levels^37^. It equals the work done from the environment on the parcel to overcome inhibition to its lifting. It is defined as follows:

$$\begin{aligned} CINE=\int _{SFC}^{LFC}(T_{vp}-T_{ve}) R_{d}dln P \end{aligned}$$

where SFC is the lowest level of the parcel from where it is lifted.

SWEAT is used to evaluate the potential for severe weather. It incorporates several variables and is defined in Miller^38^ as follows:

$$\begin{aligned} SWEAT =12(T_{d850}) + 20(TT-49) + 2(F_{850}) + F_{500} +125(S+0.2) \end{aligned}$$

where TT is the total totals, defined as the sum of cross totals (CT) and vertical totals (VT) which are expressed as CT = $\hbox {T}_{d850}$–$\hbox {T}_{e500}$ and VT = $\hbox {T}_{e850}$–$\hbox {T}_{e500}$. F is the wind speed measured in knots at a specific pressure level. S is the shear, defined as the sin($\hbox {W}_{d500}$–$\hbox {W}_{d850}$) where $\hbox {W}_{d}$ is the wind direction. The suffixes 850 and 500 indicate the pressure level of 850 hPa and 500 hPa respectively. SWEAT value of 250 or beyond indicates the strong possibility of storm formation.

K-index (KI) is determined by the temperature lapse rate in vertical, and the vertical extent of low-level moisture and its amount in the atmosphere^39^. It is a measure of the thunderstorm potential. KI is given by the following equation:

$$\begin{aligned} KI = (T_{e850}-T_{e500}) + T_{d850}- (T_{e700}-T_{d700}) \end{aligned}$$

here suffix 700 indicates the 700 hPa pressure level.

## Results

The storms’ characteristics are discussed in detail for pre-monsoon and monsoon seasons. The pre-monsoon season is the period between 1 May and monsoon onset dates for the year 2016 (21 June, 20 June, 19 June, and 17 June at Lucknow, Patna, Bhopal, and Nagpur respectively^40^) and the monsoon period is from the monsoon onset date till monsoon withdrawal date for the year 2016 (12 October at Lucknow, and 14 October for Patna, Bhopal, and Nagpur; IMD report^40^). Total 5793, 14346, 11923, and 7910 radar volume scans are available at Lucknow, Patna, Bhopal, and Nagpur respectively. Total 1555 (17), 1592 (717), 4477 (836), and 303 (318) monsoonal (pre-monsoonal) storms are identified at Lucknow, Patna, Bhopal, and Nagpur respectively (Table 1). This study contains results in four parts. In the next section, we present the environmental conditions in monsoon and pre-monsoon seasons which are also part of storm characteristics.

### Environmental conditions

**Figure 3:**
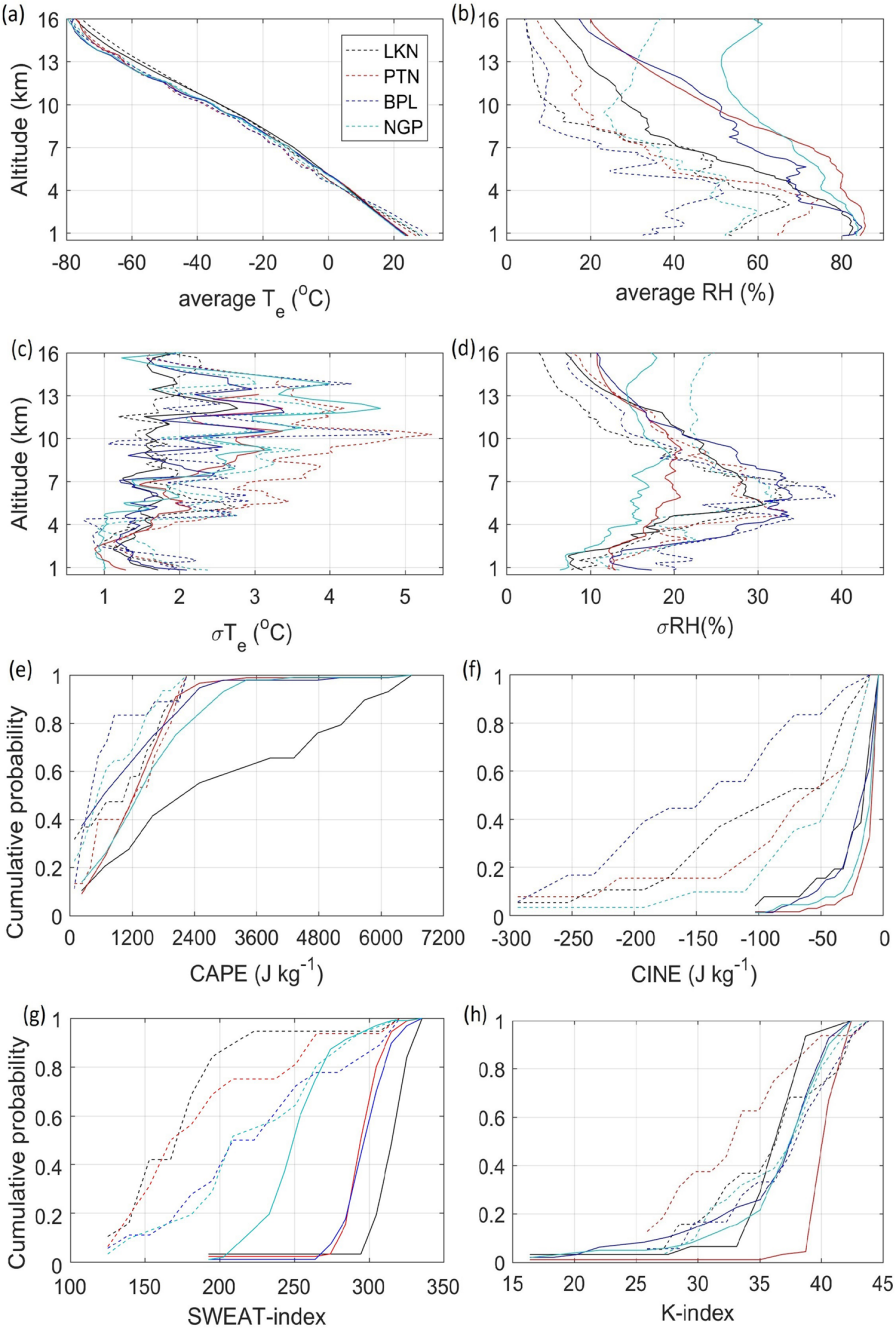
The mean (**a**,**b**) and 1-standard deviation ($\sigma$) (**c**,**d**) profiles of environmental temperature ($\hbox {T}_e$) and relative humidity (RH). The data are based on 12 UTC (1730 IST) sounding. Average environmental conditions over each radar location. The parameters shown are cumulative probability distributions of (**e**) convective available potential energy (CAPE), (**f**) convective inhibition energy (CINE), (**g**) severe weather threat (SWEAT) index, and (**h**) K-index (KI). The data for the pre-monsoon and monsoon seasons are shown in dashed and solid curves, respectively.

In this section, we present the differences in the environmental conditions observed during monsoon and pre-monsoon seasons based on the IMD sounding data. The profiles of average and standard deviation of $\hbox {T}_e$ and RH are shown in Fig. 3. The sounding data are available at two times instants 00 UTC (0530 IST) and 12 UTC (1730 IST) but only 12 UTC is considered as the convection peaks in the afternoon hours. It can be seen that the average $\hbox {T}_e$ profiles are similar but have a minute difference above and below 4 km altitude (Fig. 3a). Such differences are exhibited clearly in the profile of the standard deviation of $\hbox {T}_e$ ($\sigma \hbox {T}_e$) within the limits of 1–5 $^{\circ }$C (Fig. 3c). Profiles of $\hbox {T}_e$ and $\sigma \hbox {T}_e$ suggest that the lower troposphere ($<4$ km) is relatively cooler than the remaining tropospheric levels in monsoon season compared to pre-monsoon season. The profiles of RH and its standard deviation are shown in Fig. 3b,d. It is found that the monsoon season is relatively more humid (RH$>68\%$ at or below 4 km) than the pre-monsoon season. It is due to the winds during the southwest monsoon season which brings more moisture to the Indian land. Figure 3e–h shows the cumulative probability distributions of environmental parameters and indices. The CAPE values are larger in the monsoon season compared to the pre-monsoon season (Fig. 3e). All the CAPE populations in the pre-monsoon season are limited to 2400 J $\hbox {kg}^{-1}$. In the monsoon season, about 52%, 9%, 16%, and 25% samples observed at Lucknow, Patna, Bhopal, and Nagpur respectively exceed CAPE of 2400 J $\hbox {kg}^{-1}$. About 96–98% of CINE values limit to − 100 J $\hbox {kg}^{-1}$ in monsoon season for all locations while in pre-monsoon, only 42%, 23%, 55%, and 9% samples remain within − 100 J $\hbox {kg}^{-1}$. CINE values sometimes exceed − 280 J $\hbox {kg}^{-1}$ (Fig. 3f). The SWEAT$>250$ and KI$>30$ indicate the favourable conditions for thunderstorm formation. About 96%, 97%, 98%, and 48% of the populations at Lucknow, Patna, Bhopal, and Nagpur respectively have SWEAT more than 250 in monsoon season while in the pre-monsoon season, these are about 5%, 18%, 28%, and 38% respectively (Fig. 3g). About 93%, 98%, 86% and 92% of the populations have the KI value more than 30 in monsoon season, while about 84%, 62%, 83%, and 87% of the populations have KI value more than 30 in pre-monsoon season (Fig. 3h). These SWEAT and KI values confirm the possibility of intense clouds formation.

### Storm characteristics

**Figure 4:**
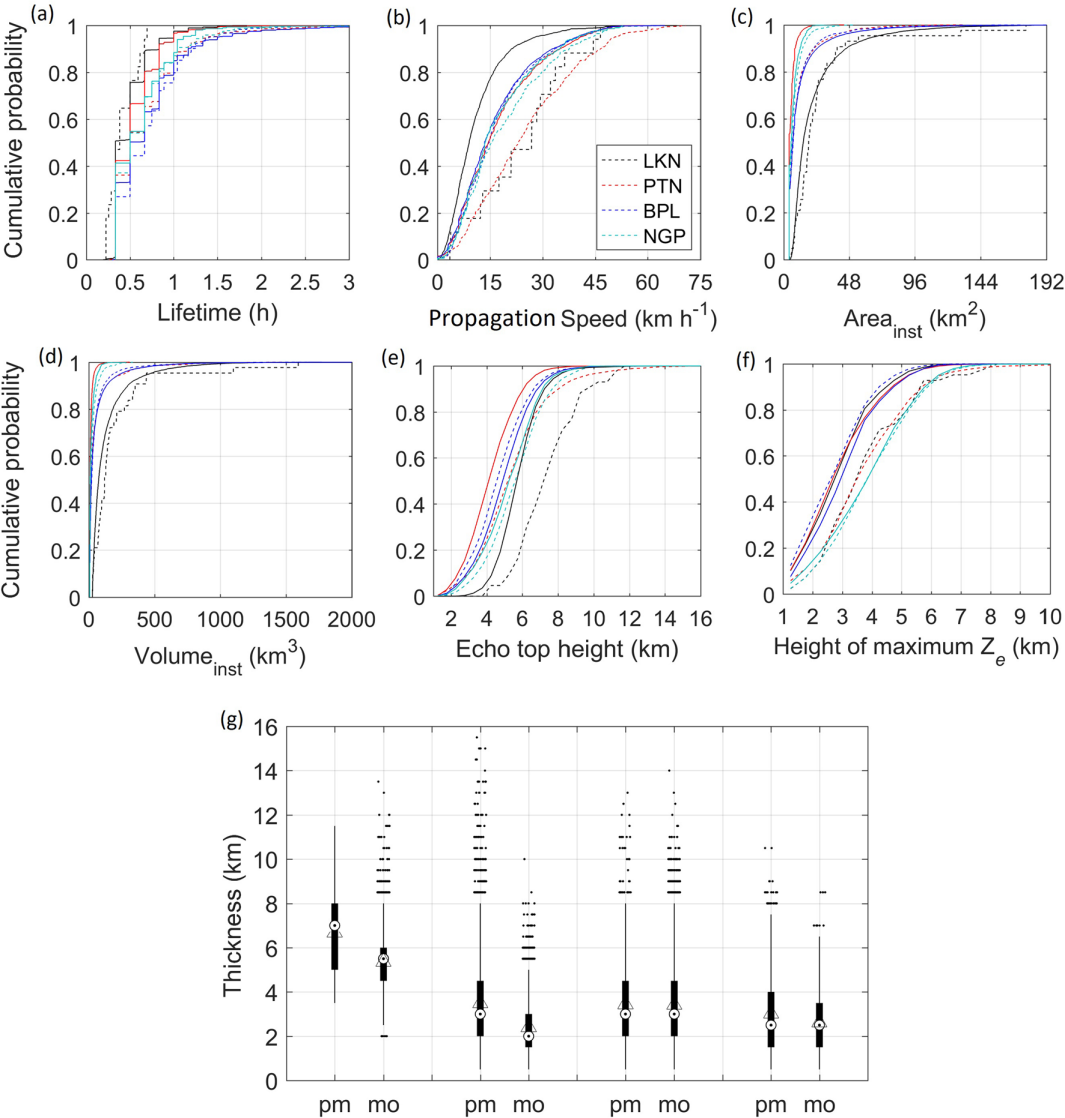
The cumulative probability distributions of (**a**) lifetime, (**b**) propagation speed, (**c**) instantaneous area, (**d**) instantaneous volume, (**e**) echo top height, and (**f**) height of maximum $\hbox {Z}_{ e}$ of storms. The data corresponding to the pre-monsoon and monsoon seasons are shown by dashed and solid curves respectively. (**g**) Distributions of instantaneous thicknesses of storms during monsoon and pre-monsoon seasons. The boxplot includes a thick bar of inter-quartile range (IQR) of data and median shown by the symbol of the black dot within a white filled circle. The upper and lower extremities of thin vertical lines are the limits of 1.5 $\times$ IQR above and lower side of the 75th and 25th percentile of the data. The symbol ‘triangle’ onto the boxplot denotes the mean value of the distribution. The symbols of the black dot are the outliers in the distributions. Here, abbreviations ‘pm’ and ‘mo’ refer to the pre-monsoon and monsoon seasons respectively.

Figure 4 shows the probability distributions of duration, propagation speed, instantaneous area, and instantaneous volume, echo top height and height of maximum $\hbox {Z}_{ e}$ of the storms. About 75–95% of the storms have a lifetime of 1 hour or less while a few exceed 2.5 h (Fig. 4a). At Lucknow, the durations of these storms do not exceed half an hour in the pre-monsoon season. The propagation speed of the storm varies between 5 and 60 km $\hbox {h}^{-1}$ in both the seasons (Fig. 4b). About 99%, 90%, 95%, and 88% of the storms identified at Lucknow, Patna, Bhopal, and Nagpur respectively have propagation speed of 30 km $\hbox {h}^{-1}$ or less in monsoon season while pre-monsoon season, these numbers are 47%, 79%, 90%, and 82%. Storms at Lucknow propagate faster ($>30$ km $\hbox {h}^{-1}$) while the slow propagating storms are observed at Bhopal in the pre-monsoon season. It is also found that the monsoonal storms propagate slower than pre-monsoonal storms. About 89–99% of the monsoon storms and 89–97% of pre-monsoonal storms at Patna, Bhopal, and Nagpur respectively have an instantaneous area of 20 $\hbox {km}^2$ or less (Fig. 4c). At Lucknow, the area of about 33–37% of the storms exceeds 20 $\hbox {km}^2$ in both seasons. About 56%, 99%, 84%, and 99% of monsoonal storms and 33%, 87%, 91%, and 96% of the pre-monsoonal storms have an instantaneous volume of 100 $\hbox {km}^3$ or less at Lucknow, Patna, Bhopal, and Nagpur respectively (Fig. 4d). The ETHs of storms lie between 2 and 14 km while the height of maximum $\hbox {Z}_{ e}$ lies between 1.5 km and 10 km (Fig. 4e,f). Lucknow stands out distinct among all stations which have 80% storms with ETHs of 6 km or beyond. Else all the locations share similar distributions of ETHs. About 85–95% of monsoonal storms and 69–91% of the pre-monsoonal storms have a height of maximum $\hbox {Z}_{ e}$ less than 5 km except for Nagpur where the height of maximum $\hbox {Z}_{ e}$ is found less than 5 km for 74% (71%) of monsoonal (pre-monsoonal) storms. The distributions of thicknesses of storms in monsoon and pre-monsoon seasons are shown in Fig. 4g. It lies within a wide range of 0.5–16 km. Both mean and median thicknesses lie between 2 and 4 km in both seasons except at Lucknow, where it is found between 5 km (7 km) in the pre-monsoon (monsoon) season.

### RERV and ATI relationships

**Figure 5:**
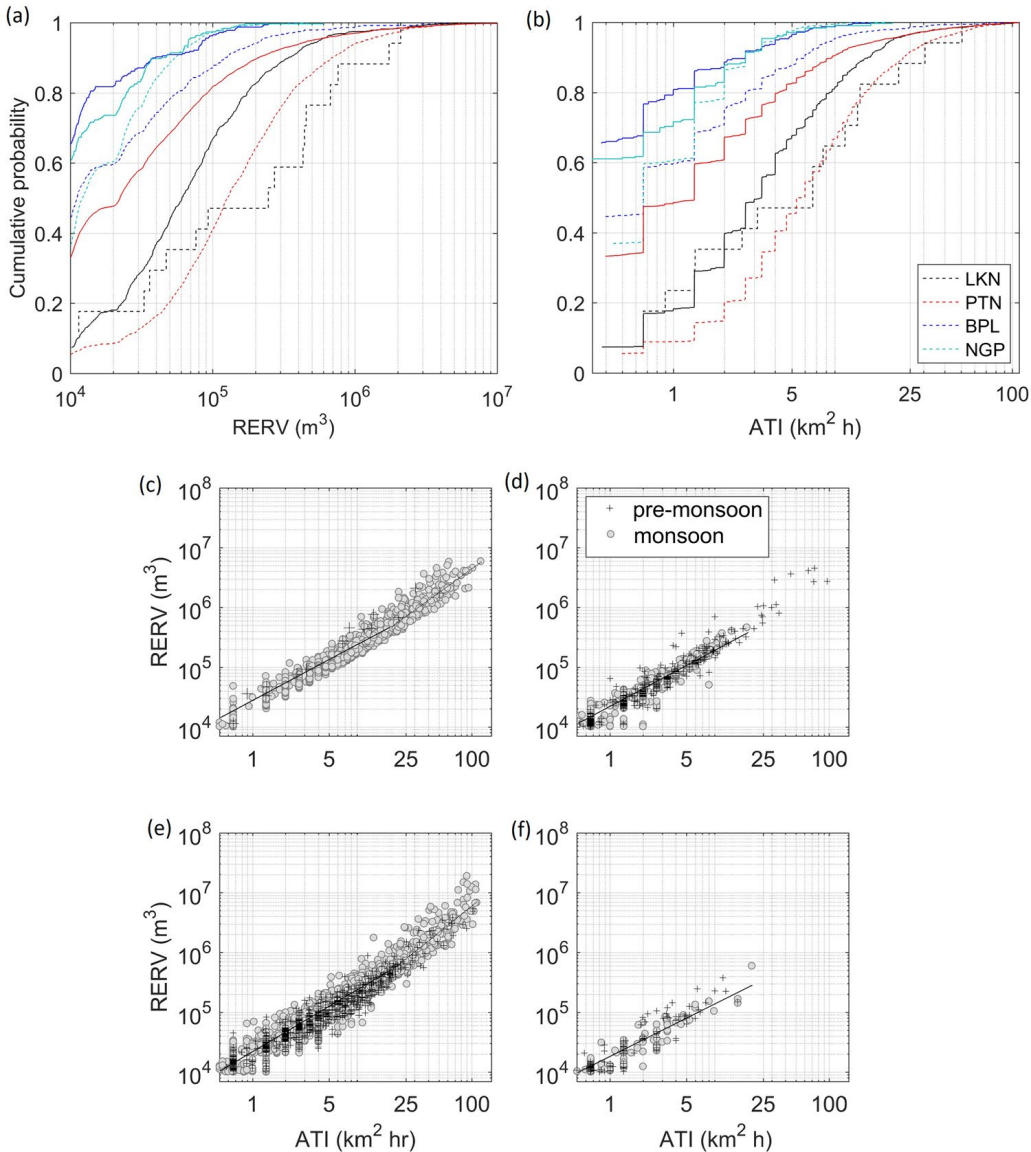
Cumulative probability distributions of (**a**) radar estimated rain volume (RERV) and (**b**) area-time integral (ATI). The dashed and solid curves indicate the pre-monsoon and monsoon season respectively. RERV versus ATI at (**c**) Lucknow, (**d**) Patna, (**e**) Bhopal, and (**f**) Nagpur. Straight lines show best-fit lines to scatter of RERV and ATI at the log–log scale. The solid and dashed best-fit lines correspond to ATI $\le20$ $\hbox {km}^2$ h and ATI $>20$ $\hbox {km}^2$ h, respectively. The data corresponding to pre-monsoon and monsoon seasons are shown by symbols ‘+’ and ‘o’ respectively.

On average, to assess how much precipitation results from an individual storm during its lifetime of a specific size, we show the relationships between RERV and ATI at each location (Fig. 5). Total 1471 (15), 1007 (398), 2991 (775), and 119 (110) RERV-ATI pairs are obtained at Lucknow, Patna, Bhopal, and Nagpur, in monsoon (pre-monsoon) season. Here, RERV-ATI pairs are less than the total number of storms because RERV is calculated at 1.5 km altitude CAPPI and some storms do not have valid $\hbox {Z}_{ e}$ at this height. The distributions of RERV and ATI are shown in Fig. 5a,b. The range of RERV and ATI lies between 10$^4$–10$^7$ $\hbox {m}^3$ and 1–100 $\hbox {km}^2$ h respectively. About 50% of the storms at Lucknow and Patna in pre-monsoon season have RERV$>10^5$ $\hbox {m}^3$ and ATI$>15$ $\hbox {km}^2$ h respectively which are at least 30% higher than Bhopal and Nagpur. The scatter plots between RERV and ATI are shown in Fig. 5c–f. Both b and K are the least at Nagpur compared to other locations, suggests that the rain volume resulted from 40-dBZ storms observed at Nagpur is less. It may be noted that the coefficient ‘b’ for ATI $\le 20$ $\hbox {km}^2$ h and ATI $>20$ $\hbox { km}^2$ h are different at Lucknow and Bhopal (Table 2). The values of exponent ‘b’ and coefficient ‘K’ for cases (1) ATI $\le 20$ $\hbox {km}^2$ h and (2) ATI $>20 \hbox { km}^2$ h are given in Table 2. For larger ATIs ($\sim$ 25–100 $\hbox {km}^2$ h), rain volume is estimated in a range between 2 $\times$ 10$^5$ to 2 $\times$ 10$^7$ $\hbox {m}^3$. In the pre-monsoon season, the characteristics (e.g., b, K, Pearson correlation coefficients-PCC) of RERV-ATI relationships are found similar to that obtained in monsoon season (Table 2). In the pre-monsoon season, ATI values are dominated below 20 $\hbox {km}^2$ h, only a few of them reach higher. On the contrary, at Patna, few of the storms attain higher ATI (25 $\hbox {km}^2$ h) and RERV (10$^6$ $\hbox {m}^3$). The scatter plots between RERV-ATI for pre-monsoon and monsoon season seem to be similar. Whether they belong to the same populations or from different, to check this hypothesis, we have performed the two-sample Kolmogorov–Smirnov test (KS-test) over RERV values in both seasons. It is found that at Lucknow, KS-test accepts the hypothesis of a similar population at 87% confidence interval, else at other locations, it rejects the hypothesis. It depicts that the characteristics of RERV-ATI pairs at Lucknow in pre-monsoon and monsoon season are found similar.

**Table 2:** Properties of RERV-ATI relationships and Pearson correlation coefficient (PCC) at 99% significant interval. Here, K and b are the coefficient and exponent respectively in RERV-ATI relationships.

b, K, PCC	ATI	Lucknow	Patna	Bhopal	Nagpur
b, K (monsoon)	ATI $\le 20$	1.09, 2.01 $\times$ 10^4^	1.03, 1.89 $\times$ 10^4^	1.09, 1.91 $\times$ 10^4^	0.95, 1.72 $\times$ 10^4^
	ATI $>20$	1.24, 0.3 $\times$ 10^4^	–	1.51, 0.68 $\times$ 10^4^	–
b, K (pre-monsoon)	All	1.15, 2.83 $\times$ 10^4^	1.12, 1.85 $\times$ 10^4^	1.07, 1.9 $\times$ 10^4^	1.01, 1.79 $\times$ 10^4^
PCC (monsoon, pre-monsoon)	All	0.91, 0.9	0.95, 0.9	0.87, 0.92	0.88, 0.86

### VPRRs at different storm life phases

**Figure 6:**
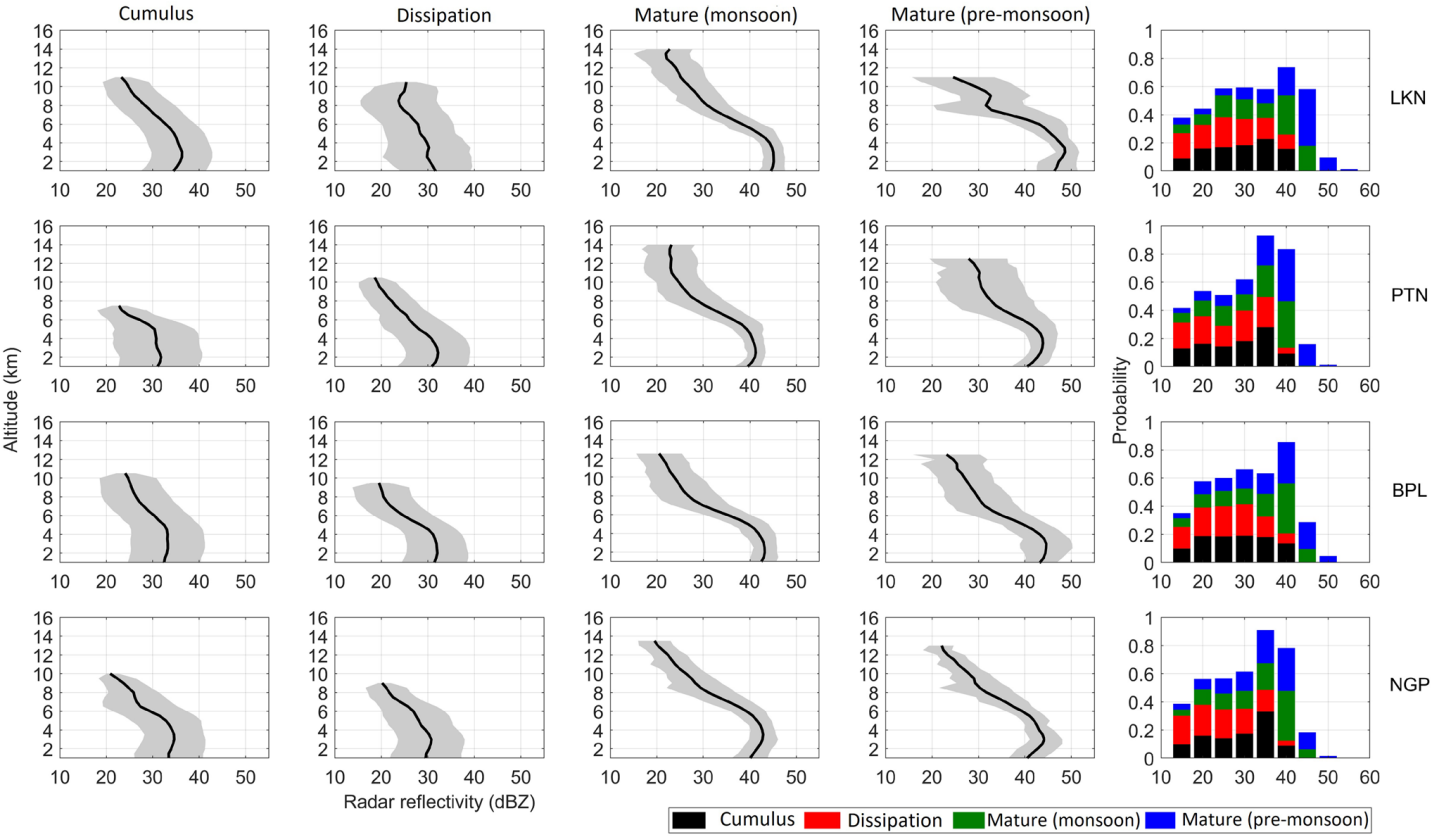
Statistics of VPRRs at cumulus, dissipation and mature phases of storm lifetime during the monsoon season. For comparison, VPRRs at mature phase are shown during the pre-monsoon season. The total numbers of VPRRs are 6948 (102), 4447 (29693), 260327 (21934) and 7392 (2083) at Lucknow, Patna, Bhopal, and Nagpur, respectively, during the monsoon (pre-monsoon) season. The thick line is the mean of all VPRRs, and the shaded region shows a 1$\sigma$ at each altitude level. The rightmost bar plots show the relative frequency of occurrence of $\hbox {Z}_{ e}$ in different phases of storm life.

The VPRRs and the probability distributions of $\hbox {Z}_{ e}$ of storms at cumulus, mature, and dissipation phases are shown in Fig. 6. The wide range of $\hbox {Z}_{ e}$ at the lowest altitude level is between 24 and 41 dBZ which increases by 2–3 dBZ more between 2 and 5 km altitudes. The dynamic range of $\hbox {Z}_{ e}$ at the cumulus phase lies between 14 and 19 dBZ. On contrary, in dissipation phase, the dynamic range remains lower (13–15 dBZ) with maximum $\hbox {Z}_{ e}$ below 40 dBZ at any altitude. Based on the mean of all VPRRs, the maximum vertical extent in cumulus (dissipation) phase are estimated as 11 km (10.5 km), 7.5 km (10.5 km), 10.5 km (9.5 km), and 10 km (9 km) km at Lucknow, Patna, Bhopal, and Nagpur, respectively. The vertical gradient of $\hbox {Z}_{ e}$ (calculated from mean of all VPRRs) in mixed-phase region (5–8 km) at cumulus (dissipation) phase are found 2.4 dBZ $\hbox {km}^{-1}$ (2.5 dBZ $\hbox {km}^{-1}$), 2.9 dBZ $\hbox {km}^{-1}$ (2 dBZ $\hbox {km}^{-1}$), 2.7 dBZ $\hbox {km}^{-1}$ (2.9 dBZ $\hbox {km}^{-1}$), and 2.5 dBZ $\hbox {km}^{-1}$ (2.2 dBZ $\hbox {km}^{-1}$) at Lucknow, Patna, Bhopal and Nagpur, respectively. In general, VPRR is used as an indicator of strong convection and vertical $\hbox {Z}_{ e}$ gradient in the mixed-phase region may differentiate convective cells based on geographical locations in different seasons^36^. With this perspective, we compared the monsoonal and pre-monsoonal storms in their mature phase. The mature phase is chosen among all three phases because this phase is more influenced by the seasonal variations than any other life phases of storms over different geographical locations. Such differences are easily noticeable by VPRRs calculated in monsoon and pre-monsoon seasons (Fig. 6). For example, the wideness at any altitude level and vertical extents of the mean profile of VPRRs vary seasonally and geographically. At Lucknow, the vertical extents of mean VPRR in monsoon and pre-monsoon seasons are 14 km and 11 km respectively along with the wider dynamic range of $\hbox {Z}_{ e}$ ($\sim$ 20 dBZ) in the monsoon season than in pre-monsoon season ($\sim$ 12 dBZ). At Patna, vertical extents of VPRRs are 14 km and 12.5 km with a spread in $\hbox {Z}_{ e}$ with a dynamic range of 17 dBZ and 11 dBZ in monsoon and pre-monsoon seasons respectively. At Bhopal and Nagpur, the vertical extents of mean VPRR are 12.5 km (12.5) km and 13.5 km (13 km) in monsoon (pre-monsoon) seasons respectively. The dynamic ranges of the wideness of $\hbox {Z}_{ e}$ are 14 dBZ for both Bhopal and Nagpur in both the seasons. The main observation from VPRRs is the maximum $\hbox {Z}_{ e}$ limit in monsoon and pre-monsoon seasons. The dynamic range of maximum $\hbox {Z}_{ e}$ is 40–47 dBZ at 3 km in monsoon season while it is found wider (39–52 dBZ). In the mixed-phase region (5–8 km), the vertical $\hbox {Z}_{ e}$ gradients in VPRRs during the mature phase are 5.18 (3.8), 3.32 (3.76), 3.51 (4.38), 3.58 (3.6) dBZ $\hbox {km}^{-1}$ in pre-monsoon (monsoon) season. The changes in the vertical $\hbox {Z}_{ e}$ gradient in the mixed-phase region is due to the mutual contest between the updraft and downdraft speeds. This process in the mixed-phase region is governed by precipitation resulted due to fallout of the hydrometeors in the presence of low updraft speed. In this scenario, low updraft speeds enable hydrometeors to become bigger and in fallout downwards, and hence downdraft speed increases. This process results in an increase in $\hbox {Z}_{ e}$ as the diameter of liquid hydrometeor increases. Another scenario may also exist in which the stronger updraft speed brings more condensed water to the higher altitudes while hydrometeors increase in size and fallout of hydrometeors occur due to large downdrafts dominants over updrafts. In both of these processes, the phase changes from liquid to ice (or vice versa) and size changes of hydrometeors due to aggregation, accretion, and deposition. During this microphysical development, the Wegener–Bergeron–Findeisen process holds well^41–43^. The $\hbox {Z}_{ e}$ distributions of all the VPRRs corresponding to each life phase of the storm are prepared (Fig. 6). It is found that about 40–50% $\hbox {Z}_{ e}$ values ($>40$ dBZ) in $\hbox {Z}_{ e}$ distributions are found at the mature phase during both monsoon and pre-monsoon seasons. The cumulus and dissipation phases are dominated by echoes less than 35 dBZ. The sharp decline in the $\hbox {Z}_{ e}$ values is seen at cumulus and dissipation phases as approaches towards the higher limit (which rarely exceeds 40 dBZ) however at the mature phase, $\hbox {Z}_{ e}$ distributions peak at 40 dBZ which extend beyond 45 dBZ.

## Discussion

The formation of storms in monsoon and pre-monsoon seasons is influenced by the environmental conditions which vary with geographical diversity. Based on the $$\hbox {T}_e$$ and $$\sigma$$$$\hbox {T}_e$$ profiles, it is found that the lower troposphere is cooler in monsoon season compared to pre-monsoon season. RH and $$\sigma$$RH profiles suggest that the monsoon season is more humid than the pre-monsoon season as also reported by Parker et al.^44^. The lower CINE and higher CAPE observed in humid monsoon season support the formation of storms while large CINE and lower CAPE in dry pre-monsoon season inhibit the storm formation. These large values of CAPE suggest the presence of an elevated mixed layer (2–4 km) and an unstable environment with strong convective updrafts^1,44^. The SWEAT and KI provide the evidence of potential available for storm formation in both seasons but dominantly in monsoon season. The lifetime of 75% of the storms is one hour or less and a few remain in existence for more than 2.5 h. Depending on the environmental and microphysical processes (CAPE, CINE, updraft speed, accretion, aggregation, deposition, the fallout of hydrometeors, etc.) along with the merging and splitting of the storms, their lifetimes are decided. Over Australia, the propagation speed for 30-dBZ storms is found between 5 and 100 km $$\hbox {h}^{-1}$$ with a mean of 28 km $$\hbox {h}^{-1}$$^45^. Over Belgium, Goudenhoofdt and Delobbe^14^ found that the speed distribution of 40-dBZ storms (with the volume of 10 $$\hbox {km}^3$$) peaks at 20 km $$\hbox {h}^{-1}$$ with a maximum $$\sim$$ 100 km $$\hbox {h}^{-1}$$. In the present study, the propagation speed lies between 5 and 70 km $$\hbox {h}^{-1}$$ for all locations. The similar range of the propagation speed is also found by Sindhu and Bhat^19^ at Delhi, a land region located in Northwest India. It is found that the pre-monsoonal storms propagate faster than that observed in monsoon season. These individual 40-dBZ storms can move alone or in the cluster depending on their merging and splitting during convective cloud lifetime, in which those are embedded. Hence, they can follow the movements of convective clouds. The storm propagation can also be influenced by the mean tropospheric winds which can be followed by the individual storms (or convective clouds). About 89–90% of the storms have an area of 20 $$\hbox {km}^2$$ with a maximum of 144 $$\hbox {km}^2$$ while 20% storms at Lucknow exceed 20$$\hbox {km}^2$$. The areas of 5–10% of the 40-dBZ storms at New Delhi found beyond 144 $$\hbox {km}^2$$, among them, few exceed 250 $$\hbox {km}^2$$^19^. The distributions of the maximum height of 35 and 40 dBZ peaks of storms are found at 10 and 15.5 km respectively in monsoon seasons over Darwin, Australia^13^. In the present study, ETHs of storms lie between 2 and 14 km with a height of maximum $$\hbox {Z}_{ e}$$ between 1.5 km and 10 km. The seasonal differences in thickness distribution are larger at Lucknow and Patna compared to Bhopal and Nagpur. The mean thickness in premonsoon storms is 1–1.5 km more than monsoonal storms at Lucknow and Patna. The echo top height is decided by the level of neutral buoyancy while echo base height is determined by the initial amount of the moisture in a rising parcel. Cloud forms when water vapor gets condensed, as decided by $$\hbox {T}_e$$ and $$\hbox {T}_d$$ profiles. While stronger updraft speeds can bring more condensed water to the higher altitudes. At Lucknow and Patna, the low-level mean RH is about 65–75% with a relatively lower mean and standard deviation of T_*e*_ compared to Bhopal and Nagpur. About 50% of pre-monsoonal storms at Lucknow and Patna again show that have RERV$$>10^5$$ $$\hbox {m}^3$$ with ATI$$>15$$ $$\hbox {km}^2$$ h. These numbers of storms are very less ($$<10\%$$) in the case of Bhopal and Nagpur. The RERV-ATI populations in monsoon and pre-monsoon seasons at Lucknow pass the hypothesis using KS-test that they come from the same populations while at other locations, the hypothesis is rejected. The seasonal similarity at Lucknow is unique among all the locations. The usefulness of the RERV-ATI analysis is about knowing the rain amount resulted from the storms during its lifetime. If the rain amount from one storm is known, then an estimate of total rain amount can be inferred from several storms in a season/month. In the present analysis, the range of the RERV is between 2 $$\times$$ 10$$^5$$ and 2 $$\times$$ 10$$^7$$ $$\hbox {m}^3$$. If a location experiences more than 200 storms in a season where each storm has the capability of RERV of more than 2 $$\times$$ 10$$^7$$ $$\hbox {m}^3$$, the total rain amount results from those storms is enough to surpass a dam of general containing capacity of 4 $$\times$$ 10$$^9$$ $$\hbox {m}^3$$. For example, Tehri Dam has a total capacity of $$\sim$$ 4 $$\times$$ 10$$^9$$ $$\hbox {m}^3$$ (http://cwc.gov.in/national-register-large-dams) and such dams can be easily overflooded by more than such 200 storms. Hence information derived from RERV-ATI pairs have an important implication in real-world problems. At Lucknow during the pre-monsoon season, the higher echo top heights and larges RERVs suggest the high $$\hbox {Z}_{ e}$$ at the lower altitude levels. Heymsfield et al.^46^ suggested that the vertical $$\hbox {Z}_{ e}$$ gradient in the mixed-phase region is sensitive to the small perturbations in updraft speed. However, the competition between updraft speed and the fallout of the hydrometeors decides the vertical $$\hbox {Z}_{ e}$$ gradients in the mixed-phase region. On the other hand, the relation between CAPE and updraft speed is complex^37,46^. The vertical extent of VPRRs can be discussed in terms of CAPE, CINE, and entrainment rate. Large CAPE and low CINE values can result in a high vertical extent of the storm. However, entrained air from the surrounding environment into the parcel can diminish the vertical extent of the storm. There is a possibility that larger echo top heights in pre-monsoon season may be due to the lower entrainment rates. However to prove this hypothesis, we do not have sufficient observations to estimate the entrainment rates. The larger CAPE, SWEAT, KI, and smaller CINE are found in monsoon seasons at all locations which can be inferred as the presence of strong convection to form many storms compared to pre-monsoon season. The minute difference in $$\hbox {Z}_{ e}$$ ($$\sim$$1 dBZ) in mean of all VPRRs below 4 km calculated from monsoonal storms abide by these environmental conditions (at Lucknow, Patna, and Bhopal) while this difference is enhanced ($$\sim$$ 4 dBZ) in pre-monsoon season. Due to this, the mean of all VPRRs gets flattened shape below 4 km. Another striking feature is observed at the mature phase in which, a larger magnitude of $$\hbox {Z}_{ e}$$ (30–45 dBZ) in mean of all VPRRs exist throughout the mixed-phase region in the pre-monsoon season compared to with monsoon season. For an individual storm, it exceeds 51 dBZ. It suggests that the fallout of hydrometeors dominates over updraft while they remain there for a longer time in the mixed-phase region. Using NOAA-TOGA C-band radar data at Darwin (Australia), Zipser and Lutz^36^ stated the vertical $$\hbox {Z}_{ e}$$ gradients between 0$$^{\circ }$$ and − 20 $$^{\circ }$$C (approximately 5–9 km) are found as 1.5 and 3.5 dBZ $$\hbox {km}^{-1}$$ for midlatitude continental and tropical continental cases respectively. Using WSR-88D at League City (USA), Toracinta et al.^47^ found that median VPRRs have the vertical $$\hbox {Z}_{ e}$$ gradient of 2 dBZ $$\hbox {km}^{-1}$$. Using TRMM data, Bhat and Kumar^48^ studied vertical structures of intense convective cells (identified at 3 km using $$\hbox {Z}_{ e}$$ threshold range 39.5–41.5 dBZ) over Indian land and oceanic regions. They showed that the vertical $$\hbox {Z}_{ e}$$ gradients lie between 3 and 3.5 dBZ $$\hbox {km}^{-1}$$ over land regions during the Indian summer monsoon season. Using S-band radars over India, Sindhu and Bhat^2^ identified the convective echoes in monsoonal cloud systems and found that the vertical $$\hbox {Z}_{ e}$$ gradients between 5–8 km altitudes are in the range of 3.1–4.5 dBZ $$\hbox {km}^{-1}$$. In the present study, the vertical $$\hbox {Z}_{ e}$$ gradients lie in the ranges between 2 dBZ $$\hbox {km}^{-1}$$ and 4.4 dBZ $$\hbox {km}^{-1}$$ in monsoon season. The estimates of vertical $$\hbox {Z}_{ e}$$ gradients in the present study are comparable with the previous findings available at land regions.

## Conclusions

The storm characteristics based on 40-dBZ of radar reflectivity threshold are studied in detail during monsoon and pre-monsoon seasons using Doppler weather radar data at Lucknow, Patna, Bhopal, and Nagpur. The conclusions drawn based on this study are as follows:

1. The environmental indices (e.g., CINE, CAPE, SWEAT, KI) in monsoon season are found more favourable for strong convection than pre-monsoon season which strengthens the possibility of formation of more storms.
2. The lifetimes of the 75–95% of monsoonal and pre-monsoonal storms are estimated 1 h or less. Few storms exceed lifetime of 2.5 h. The propagation speed of storms lies between 5 and 60 km $\hbox {h}^{-1}$. At Lucknow and Patna, fast propagating storms are more in pre-monsoon season and compared to Bhopal and Nagpur. The area and volume of the storms lie between 4–184 $\hbox {km}^2$ and 8–1600 $\hbox {km}^3$ respectively. The echo top heights and the height of maximum $\hbox {Z}_{ e}$ of storms are found between 2–14 km and 1.5–10 km respectively. The storm thicknesses lie between 0.5–16 km while the mean values of the thickness populations in both the seasons are estimated between 2 and 7 km.
3. The RERV and ATI lie in ranges10$^4$–10$^7$ $\hbox {m}^3$ and 1–100 $\hbox {km}^2$ h respectively in both monsoon and pre-monsoon seasons. The results from the two-sample KS-test applied at RERV populations in both the seasons suggest that monsoonal and pre-monsoonal storms have similar precipitation characteristics at Lucknow at 87% confidence interval while at other locations, RERV-ATI pairs are independent to each other. The RERV-ATI pairs are found important to assess the capability of storms in terms of the total precipitation resulted from a series of storm events in a season.
4. In mixed-phase region, the vertical $\hbox {Z}_{ e}$ gradients in mean of all VPRRs lie within ranges 2.4–2.9 dBZ $\hbox {km}^{-1}$, 3.6–4.4 dBZ $\hbox {km}^{-1}$ and 2–2.9 dBZ $\hbox {km}^{-1}$ at cumulus, mature and dissipation phases of storms in monsoon season. At mature phase, these gradients lie between 3.3 dBZ $\hbox {km}^{-1}$and 5.2 dBZ $\hbox {km}^{-1}$ in pre-monsoon season. At mature phase, the dynamic range of $\hbox {Z}_{ e}$ in mean of all VPRRs below 4 km is larger ($\sim$ 4 dBZ) in pre-monsoon season which is found marginal ($\sim$ 1 dBZ) in monsoon season. It results into the more flattened mean of all VPRRs at Lucknow, Patna, and Bhopal. In mixed-phase region at the mature phase, $\hbox {Z}_{ e}$ values in mean of all VPRRs lie between 30–45 dBZ in the pre-monsoon which is larger than that found in monsoon season.

The understanding of storm-scale characteristics has direct implications in short-range weather forecasting as well as improvement in convective parametrization schemes. Our findings provide the concrete evidence of total precipitation resulted from an individual storm event during its lifetime.

## References

[1] Houze RA (2004). Mesoscale convective systems. Rev. Geophys..

[2] Sindhu KD, Bhat GS (2018). Characteristics of monsoonal precipitating cloud systems over the Indian subcontinent derived from weather radar data. Q. J. R. Meteorol. Soc..

[3] Houze J, Robert A, Cheng C-P (1977). Radar characteristics of tropical convection observed during gATE: Mean properties and trends over the summer season. Monthly Weather Rev..

[4] Gill AE (1980). Some simple solutions for heat-induced tropical circulation. Q. J. R. Meteorol. Soc..

[5] Zhang Z, Krishnamurti TN (1996). A generalization of Gill’s heat-induced tropical circulation. J. Atmos. Sci..

[6] Narasimha R, Diwan SS, Duvvuri S, Sreenivas KR, Bhat GS (2011). Laboratory simulations show diabatic heating drives cumulus-cloud evolution and entrainment. Proc. Natl. Acad. Sci..

[7] Arakawa A, Schubert WH (1974). Interaction of a cumulus cloud ensemble with the large-scale environment, part I. J. Atmos. Sci..

[8] Cohen C (2000). A quantitative investigation of entrainment and detrainment in numerically simulated cumulonimbus clouds. J. Atmos. Sci..

[9] CIMO. Guide to meteorological instruments and methods of observation; secretariat of the WMO. Part II observing methods. *WMO* **8**, II.9–6–12 (2008).

[10] Steiner M, Houze J, Robert A, Yuter SE (1995). Climatological characterization of three-dimensional storm structure from operational radar and rain gauge data. J. Appl. Meteorol..

[11] Dixon M, Wiener G (1993). Titan: thunderstorm identification, tracking, analysis, and nowcasting-a radar-based methodology. J. Atmos. Ocean. Technol..

[12] Potts RJ, Keenan TD, May PT (2000). Radar characteristics of storms in the Sydney area. Monthly Weather Rev..

[13] May PT, Ballinger A (2007). The statistical characteristics of convective cells in a monsoon regime (Darwin, northern Australia). Monthly Weather Rev..

[14] Goudenhoofdt E, Delobbe L (2013). Statistical characteristics of convective storms in Belgium derived from volumetric weather radar observations. J. Appl. Meteorol. Climatol..

[15] Caine S (2013). Statistical assessment of tropical convection-permitting model simulations using a cell-tracking algorithm. Monthly Weather Rev..

[16] Shah S, Notarpietro R, Branca M (2015). Storm identification, tracking and forecasting using high-resolution images of short-range x-band radar. Atmosphere.

[17] Novo S, MartÃnez D, Puentes O (2014). Tracking, analysis, and nowcasting of cuban convective cells as seen by radar. Meteorol. Appl..

[18] Davini P, Bechini R, Cremonini R, Cassardo C (2012). Radar-based analysis of convective storms over Northwestern Italy. Atmosphere.

[19] Sindhu K. D, Bhat G. S (2019). Storm characteristics and precipitation estimates of monsoonal clouds using c-band polarimetric radar over northwest india. Theoret. Appl. Climatol..

[20] Kannemadugu H (2019). Seasonal characteristics of atmospheric boundary layer and its associated dynamics over central india. Asia Pac. J. Atmos. Sci..

[21] Turner AG (2020). Interaction of convective organization with monsoon precipitation, atmosphere, surface and sea: The 2016 incompass field campaign in india. Q. J. R. Meteorol. Soc..

[22] Iguchi T, Kozu T, Meneghini R, Awaka J, Okamoto K (2000). Rain-profiling algorithm for the TRMM precipitation radar. J. Appl. Meteorol..

[23] Hersbach H (2020). The era5 global reanalysis. Q. J. R. Meteorol. Soc..

[24] Gadgil S (2003). The Indian monsoon and its variability. Annu. Rev. Earth Planet. Sci..

[25] Houze RA, Wilton DC, Smull BF (2007). Monsoon convection in the Himalayan region as seen by the TRMM precipitation radar. Q. J. R. Meteorol. Soc..

[26] Hunt KMR, Parker DJ (2016). The movement of Indian monsoon depressions by interaction with image vortices near the himalayan wall. Q. J. R. Meteorol. Soc..

[27] IMD. Imd report 2011: Standard operating procedure for doppler weather radar-98d/s. https://dokumen.tips/documents/palam-new-delhi-standard-operating-procedure-for-doppler-weather-radar-98ds.html (2011).

[28] Booker HG (1946). Elements of radio meteorology: How weather and climate cause unorthodox radar vision beyond the geometrical horizon. J. Inst. Electr. Eng. Part I Gen..

[29] Donaldson J, Ralph J (1970). Vortex signature recognition by a Doppler radar. J. Appl. Meteorol..

[30] Fabry F (2015). Radar Meteorology: Principle and Practice.

[31] Dixon, M. Eol/ral. radx c++ software package for radial radar data. http://www.ral.ucar.edu/projects/titan/docs/radial_formats/radx.html (2014).

[32] Steiner M, Houze J, Robert A (1997). Sensitivity of the estimated monthly convective rain fraction to the choice of Z–R relation. J. Appl. Meteorol..

[33] Byers HR, Braham J, Roscoe R (1948). Thunderstorm structure and circulation. J. Meteorol..

[34] Doneaud A, Ionescu-Niscov S, Priegnitz DL, Smith PL (1984). The area-time integral as an indicator for convective rain volumes. J. Clim. Appl. Meteorol..

[35] Johnson LR, Smith PL, Vonder TH, Reinke D (1994). The relationship between Area-time integrals determined from satellite infrared data by means of a fixed-threshold approach and convective rainfall volumes. Monthly Weather Rev..

[36] Zipser EJ, Lutz KR (1994). The vertical profile of radar reflectivity of convective cells: A strong indicator of storm intensity and lightning probability?. Monthly Weather Rev..

[37] Williams E, Renno N (1993). An analysis of the conditional instability of the tropical atmosphere. Monthly Weather Rev..

[38] Miller, R. C. *Notes on analysis and severe-storm forecasting procedures of the air force global weather central* (Air Weather Service, USAF, 1972).

[39] George JJ (1960). Weather Forecasting for Aeronautics.

[40] IMD. Imd monsoon report 2016. https://metnet.imd.gov.in/imdnews/ar2016.pdf (2016).

[41] Wegener, A. *Thermodynamik der atmosphäre* 331 (Leipzig, 1911).

[42] Bergeron, T. On the physics of clouds and precipitation. In: *International Union of Geodesy and Geophysics* 156–178 (Proces Verbaux de l’Association de Météorologie, 1935).

[43] Findeisen, W. Kolloid-meteorologische Vorgänge bei Neiderschlags-bildung. *Meteor. Z.* **55**, 121–133 (1938).

[44] Parker DJ (2016). The interaction of moist convection and mid-level dry air in the advance of the onset of the indian monsoon. Q. J. R. Meteorol. Soc..

[45] Peter, J. R., Manton, M. J., Potts, R. J., May, P. T., Collis, S. M. & Wilson, L. Radar-derived statistics of convective storms in Southeast Queensland. *J. Appl. Meteorol. Climatol.* **54**, 1985–2008. 10.1175/JAMC-D-13-0347.1 (2015).

[46] Heymsfield GM, Tian L, Heymsfield AJ, Li L, Guimond S (2010). Characteristics of deep tropical and subtropical convection from nadir-viewing high-altitude airborne Doppler radar. J. Atmos. Sci..

[47] Toracinta ER, Mohr KI, Zipser EJ, Orville RE (1996). A comparison Of WSR-88D reflectivities, SSM/I brightness temperatures, and lightning for mesoscale convective systems in Texas. part I: radar reflectivity and lightning. J. Appl. Meteorol..

[48] Bhat GS, Kumar S (2015). Vertical structure of cumulonimbus towers and intense convective clouds over the South Asian region during the summer monsoon season. J. Geophys. Res. Atmos..

